# Is Animal-assisted intervention (AAI) a feasible treatment in a recovery-oriented psychiatric rehabilitation program ?

**DOI:** 10.1192/j.eurpsy.2025.2176

**Published:** 2025-08-26

**Authors:** A. Litta, D. Benazzi, P. Carbutti, A. Vacca, A. M. Nannavecchia, A. Morelli, A. M. Sisto, E. Attolino, P. Manigrasso, M. Nacci

**Affiliations:** 1 Department of Precision and Regenerative Medicine and Ionian Area (DiMePRe-J) University of Bari “Aldo Moro”, Bari; 2 Mental Health Department, ASL Taranto, Taranto; 3 EPASSS Foundation; 4 AReSS Puglia- Regional Strategic Agency for Health and Social, Bari; 5 Third Sector Organization “La coda di Ulisse”; 6 Third Sector Organization “La coda di Ulisse”, Massafra, Italy

## Abstract

**Introduction:**

Physical contact with the animal appears, on the basis of the latest research, acts as powerful calming factor on heart rate and breathing (Nose et al., 2022). Moreover, taking care of an animal stimulates a sense of responsibility and promotes empathy and kindness (Walsh et al., 2009). Studies have suggested that animals may serve as “social catalysators” involving feelings of safety and facilitation of interpersonal interactions. These effects are related to biophilia hypothesis which describes the affinity of humans to other living species (Tyssedal et al., 2023).

**Objectives:**

Our project aimed to assess effectiveness and feasibility of animal assisted intervention (AAI) in patients with psychotic spectrum disorders following a recovery-oriented psychiatric rehabilitation program.

**Methods:**

In the present study ten patients from psychiatric residential facilities belonging to the EPASSS Foundation were approached to participated in this study. Patients followed a rehabilitation project named “Animal-Mente” and originated from the collaboration of the psychiatric residential facilities belonging to the EPASSS Foundation with “La coda di Ulisse”, a Third Sector Organization (ETS) which represents the Apulian reference centre for AAI. An observational study design was followed in which the new intervention based on AAI was added to treatment as usual (usual rehabilitation intervention and/or psychopharmacological treatment). All sessions were performed following the Italian National Guidelines for animal assisted interventions and required a multidisciplinary team capable of managing the complexity of the human-animal relationship. Outcome assessments were conducted at recruitment (time 0) and after animal-assisted intervention (time 1). Outcome assessments were conducted at recruitment (time 0) and after animal-assisted intervention ( time 1). They included : Personal and Social Functioning Scale (FPS of the V.A.D.O.), Brief Rating Psychiatric Scale (BPRS), Recovery Evaluation Scale (RAS), Quality of Life Index (Q-Index), S.T.A.I.-Y questionnaire.

**Results:**

All patients followed a psychosocial rehabilitation intervention and all but one had psychopharmacological treatment in the last six months. BPRS (p-value = 0.022) and RAS scale (p-value = 0.006) showed a significant variation at time 1 compared to time 0. No one reported worsening of psychotic or other symptoms during entire program.

**Conclusions:**

Our data highlighted the feasibility of Animal-Assisted Interventions (AAI) in community mental health services. Moreover, our study underlined the opportunity of AAI in an integrative recovery oriented psychiatric rehabilitation program involving mental health department, psychiatric residential facilities and third sector organizations in a network activity.

**Disclosure of Interest:**

None Declared

